# Highly efficient transduction of primary adult CNS and PNS neurons

**DOI:** 10.1038/srep38928

**Published:** 2016-12-13

**Authors:** Evgeny Levin, Heike Diekmann, Dietmar Fischer

**Affiliations:** 1 Division of Experimental Neurology, Medical Faculty, Heinrich-Heine-University, Merowingerplatz 1a, 40225 Düsseldorf, Germany

## Abstract

Delivery and expression of recombinant genes, a key methodology for many applications in biological research, remains a challenge especially for mature neurons. Here, we report easy, highly efficient and well tolerated transduction of adult peripheral and central neuronal populations of diverse species in culture using VSV-G pseudo-typed, recombinant baculovirus (BacMam). Transduction rates of up to 80% were reliably achieved at high multiplicity of infection without apparent neuro-cytopathic effects. Neurons could be transduced either shortly after plating or after several days in culture. Co-incubation with two different baculoviruses attained near complete co-localization of fluorescent protein expression, indicating multigene delivery. Finally, evidence for functional protein expression is provided by means of cre-mediated genetic recombination and neurite outgrowth assays. Recombinant protein was already detected within hours after transduction, thereby enabling functional readouts even in relatively short-lived neuronal cultures. Altogether, these results substantiate the usefulness of baculovirus-mediated transduction of mature neurons for future research in neuroscience.

Protein overexpression and gene knockout are key technologies for the study of molecular mechanisms in life sciences. However, post-mitotic and in particular adult neurons are generally difficult to culture and particularly resistant to the delivery and expression of recombinant genes, thereby often limiting experimental approaches. Therefore, an easy and reliable method to genetically manipulate cultured neurons would be highly desirable in order to facilitate research on the molecular basis of neuronal function under normal and pathological conditions.

Despite ongoing advances in physical, chemical and electrical methods of gene delivery, primary neurons still tend to be refractory to plasmid transfection in cell culture. Although nucleofection can achieve 60–80% efficiency after optimization, this technique is mainly restricted to freshly isolated, embryonic and postnatal neurons and requires relatively expensive equipment and reagents^1^,^2^. Calcium phosphate precipitation and lipofection methods achieve at best 5–10% transfection rates and are, moreover, associated with general toxicity and transient expression^2^. Viral gene delivery systems, such as recombinant lentivirus (LV) or adeno-associated virus (AAV), may overcome the problem of low efficient gene transfer particularly into non-dividing neurons. Some studies report significant transduction rates for embryonic or postnatal cerebellar and hippocampal neurons^3^,^4^,^5^,^6^,^7^, but gene transfer into adult neurons can be considerably less efficient (5–10% for LV-transduction of adult dorsal root ganglion (DRG) neurons in our hands). Furthermore, the above-mentioned viral expression technologies have significant drawbacks, limiting their applicability for neuronal cell cultures. These shortcomings comprise cytotoxicity at high titers, risk of insertional mutations, late onset of transgene expression (notably for AAV at 5–14 days after transduction), limited insert size (<2.5 kb for AAV, 2.5–5 kb for LV) and requirement for biosafety level 2 for LV^2^.

Baculoviruses offer several advantages compared to other viral gene delivery vectors in terms of safety, high insert size capacity and ease of production and have therefore been widely used for heterologous protein expression^8^,^9^. Natural baculoviruses infect insects and cannot replicate in mammalian cells. Increased tropism can be achieved via modification of the baculoviral envelope glycoprotein gp64^10^. For example, vesicular stomatitis virus envelope G-protein (VSV-G) -pseudo-typed virions feature more efficient cell entry and transduction of a variety of mammalian cells^11^. BacMam is a genetically engineered baculovirus with VSV-G-modified capsid protein that contains a DNA cassette for transgene expression in mammalian cells, which ignore insect-specific promoters and express only mammalian promoter-driven transgenes. BacMam virus transduction is generally well tolerated without apparent cytotoxicity, even at high multiplicity of infection (MOI)^10^,^12^,^13^. In addition, baculoviruses are easy to use as they are simply incubated with cells in normal culture medium and can be handled in a biosafety level 1 facility. Therefore, the BacMam technology seemed a good candidate for gene transfer and expression in neurons^14^,^15^,^16^. To our surprise, we were unable to identify any published description of successful baculovirus-mediated transduction of adult neurons *in vitro*.

Here, we provide first evidence that primary neurons of the adult central nervous system (CNS) as well as peripheral nervous system (PNS) can be very efficiently transduced in culture using BacMam virus. Observed transduction rates markedly exceeded previously reported attempts with other gene transfer methods without obvious neuro-cytopathic effects. BacMam-induced expression of heterologous proteins was promptly initiated and proofed functional, as determined by successful cre-mediated genetic recombination and neurite outgrowth assays. Thus, this methodology provides novel experimental opportunities with cultured primary neurons, which could greatly advance future research in neuroscience.

## Materials and Methods

### Animals

All experimental animal procedures were approved by the local animal care committee (Umweltministerium NRW, Landesumweltamt, Recklinghausen) and conducted in compliance with federal and state guidelines for animal experiments in Germany. Adult (6–8 weeks old) male and female Wistar rats and mice of strains C57BL/6 and ROSA-tdTomato [C57BL/6;Cg-*Gt(ROSA)26Sor*^*tm14(CAG-tdTomato)Hz*]^*w*ere maintained on a 12 h light/dark cycle with ad libitum access to food and water. Adult, 4–8 months old, homozygous Tg(fGAP43:GFP) zebrafish^17^ were kept in the zebrafish facility of the University of Düsseldorf on a 14 h light/10 h dark cycle under standard conditions^18^. Rats were sacrificed by inhalation of CO_2_, mice by cervical dislocation and zebrafish by immersion in MS222 (0.4 mg/l) and decapitation.

### Vectors

Farnesylated EGFP (fGFP), which is targeted to the plasma membrane (kindly provided by Prof. Joost Verhaagen, Amsterdam), DsRed-Monomer (Clontech) and Cre-HA (kindly provided by Dr. Zhigang He, Boston) were directionally cloned into the Gateway pENTR 2B Dual Selection Vector (Thermo Fisher) using respective restriction enzymes. For the generation of a hyper-interleukin 6 (hIL6) and EGFP co-expression vector, an MCS-IRES-EGFP sequence was amplified using primers 5′-AATGAATTCCTCGAGCTAACGTTACTGGCCGAA-3′ and 5′-TCATTACTTGTACAGCTCGT-3′ and inserted into the pENTR 2B Gateway vector. HIL6^19^ was then directionally cloned into this modified vector. All expression constructs were transferred into the BacMam pCMV-Dest vector using Gateway LR Clonase II Enzym-Mix (Thermo Fisher) and transformed into MaxEfficiency BH10Bac cells for transposition into a bacmid according to manufacturer’s protocols. The BacMam pCMV-DEST vector contains the cytomegalovirus (CMV) promoter for high-level expression in mammalian cells, Woodchuck Hepatitis Virus Posttranscriptional Regulatory Element (WPRE) for increased duration of gene expression, SV40 polyadenylation signal for efficient transcription termination and polyadenylation of mRNA and VSG protein for viral delivery into mammalian cells (Thermo Fischer).

### Generation of recombinant baculovirus

Recombinant baculoviruses were produced using the ViraPower BacMam Expression System (Thermo Fisher) according to manufacturer’s protocols. In brief, recombinant bacmid DNA was purified and transfected into adherent Sf9 cells (Thermo Fisher) using Cellfectin reagent to generate P1 recombinant baculovirus stock. Baculoviruses were amplified by inoculation of 50 ml Sf9 suspension cultures (10^6^ cells/ml) in Sf-900 III SFM Medium supplemented with 12.5 U/ml penicillin/streptomycin (Biochrom) in 125 ml polycarbonate Erlenmeyer flasks with vent cap (Corning) with 1 ml virus stock solution and incubation at 27 °C and 130 rpm for 2–3 days. Amplified viruses were purified and concentrated by ultracentrifugation of 27 ml virus-containing supernatant underlayed with 2.7 ml sucrose solution (25% sucrose with 5 mM NaCl and 10 mM EDTA in H_2_O) in OptiSeal polypropylene tubes (Beckmann Coulter) at 80,000 g and 4 °C for 80 min. Viral pellets were re-suspended in 0.5 ml PBS and passed through 0.22 μm low protein binding sterile syringe filters (Merck Millipore). Baculovirus preparations were pre-tested on HEK293 cells (seeded at ~3–5 × 10^4^ cells per well in 96-well plates) by adding 1 μl virus per well overnight. Transduction efficiencies of ≥ 90% were regarded appropriate for further use. Otherwise, virus stock was subjected to further amplification cycles.

### Baculovirus titration

Baculoviral titers were determined using the FastPlax Titer Kit (Merck Millipore). Sf9 cells (3 × 10^4^ cells/well) were seeded into 96-well plates (Thermo Fisher) and inoculated with 20 μl serial virus dilutions (10^−3^ – 10^−7^ in duplicate) for 1 h at room temperature with gentle rocking. Thereafter, 100 μl culture medium was added to each well and cells were incubated at 27 °C for 30 h. After fixation in 4% paraformaldehyde (PFA, Sigma), transduced cells were stained with an antibody against the gp64 envelope protein and XGal staining according to the manufacturer’s protocol and quantified using an inverted microscope (Observer.D1, Zeiss). All baculoviruses used in this study had calculated titers of ~10^8^ pfu/ml.

### DRG cultures

DRG neurons were isolated from rats and mice as described previously^19^,^20^. In brief, DRG were harvested, incubated in Dulbecco’s modified Eagle medium (DMEM) containing 0.25% trypsin/EDTA (Thermo Fisher) and 0.3% collagenase type IA (Sigma) and mechanically dissociated. Cells were re-suspended in DMEM supplemented with 10% fetal bovine serum (FBS, GE Healthcare) and 500 U/ml penicillin/streptomycin (BioChrom) and 50 μl of this mixed population (containing ~100–500 DRG neurons) plated into 96-well plates coated with poly-D-lysine (0.1 mg/ml, molecular weight <300,000 Da, Sigma) and 20 μg/ml laminin (Sigma). The respective baculovirus (10 μl in PBS, ~10^6 ^pfu) was directly added 4 h after seeding and cells incubated at 37 °C and 5% CO_2_. Two-thirds of the DRG culture medium was replaced with fresh medium 16 h after transduction. After 1, 2 or 3 days in culture, cells were fixed in 4% PFA for 25 min and permeabilized in 100% methanol for 10 min. DRG neurons were identified with TUJ-1 antibody against βIII-tubulin (1:2,000, BioLegend) and in some cases co-stained with either isolectin B4 (FITC-conjugated, 1:40, L2895, Sigma) or an antibody against 200 kD neurofilament (1:1000, ab7795, Abcam). All experiments were performed in duplicate with three replicate wells for each experimental group. Percentages of fGFP- or tdTomato-positive, transduced DRG neurons in relation to TUJ-1, isolectin B4 and neurofilament counts, respectively, were quantified using a fluorescent microscope (Observer.D1, Zeiss) and presented as means ± SEM. Significances of intergroup differences were evaluated using one-way analysis of variance (ANOVA) with Holm-Sidak post hoc test.

### Retinal cultures

Retinal cultures from rats, mice and zebrafish were prepared as described previously^21^,^22^. In brief, retinae were dissected from eyecups and digested in DMEM containing papain (16.4 U/ml for rat and zebrafish, 10 U/ml for mouse retinae, respectively; Worthington) and L-cysteine (0.3 mg/ml for rat and zebrafish, 0.2 mg/ml for mouse retinae, respectively; Sigma) at 37 °C for 30 minutes (rat and mice retinae) or at room temperature for 40 minutes (zebrafish retinae). Rat and mice retinae were triturated and washed by centrifugation in 50 ml DMEM (7 min at 900 g for rat and 7 min at 500 g for mouse retinae). Retinal pellets were re-suspended in DMEM (6.5 ml/rat retina, 1.5 ml/mouse retina) containing B27-supplement (1:50, Thermo Fisher) and 200 U/ml penicillin/streptomycin to contain ~1–2 × 10^3^ retinal ganglion cells (RGC)/ml. Zebrafish retinae were rinsed with L15/salt solution (12.5% salt solution: 10 mM D-glucose, 1.26 mM CaCl2, 32 mM Hepes, pH 7.5/87.5% L15; Thermo Fisher) prior to trituration in 2 ml fish medium (2% FBS, 0.2 mg/ml penicillin/streptomycin in L15/salt solution). Cells were seeded into 96-well plates (50 μl, for the determination of transduction rates) or 4-well plates (300 μl, for outgrowth assays) coated with poly-D-lysine (0.1 mg/ml, molecular weight <300,000 Da, Sigma) and 20 μg/ml laminin (Sigma). The respective baculovirus was directly added 4 h after seeding and cells incubated at 37 °C and 5% CO_2_ (rat and mice RGCs) or at 27 °C (zebrafish RGCs). Generally, 10^6 ^pfu (10 μl in PBS) were used per well for the determination of transduction rates, 5 × 10^5 ^pfu each for co-transduction and 10^5 ^pfu (1 μl in PBS) for neurite outgrowth assays. Two-thirds of the RGC culture medium was replaced with fresh medium 16 h after transduction. After 1–6 days in culture, cells were fixed in 4% PFA for 25 min and in case of subsequent immunocytochemistry permeabilized in 100% methanol for 10 min. Rat and mice RGCs were identified with TUJ-1 antibody (1:2,000; BioLegend), while zebrafish RGCs express EGFP^22^. All experiments were performed in duplicate with at least three replicate wells per experimental group. Percentages of GFP- or DsRed-positive, transduced RGCs in relation to TUJ-1 and EGFP, respectively, were quantified at 1, 2 and 3 days for rat and mice RGCs and 4 and 6 days for zebrafish RGCs using a fluorescent microscope (Observer.D1, Zeiss) and presented as means ± SEM. In addition, the number of surviving RGCs was quantified per well. Significances of intergroup differences were evaluated using one-way analysis of variance (ANOVA) with Holm-Sidak post hoc test or Student’s t-test. For neurite outgrowth assays, RGCs at 4 days in culture with neurites longer than twice the soma diameter were photographed using a fluorescent microscope (200×, Observer.D1, Zeiss) and neurite length was determined using ImageJ software. In addition, βIII-tubulin-, DsRed- or GFP-positive RGCs were quantified per well. Average neurite length for transduced and non-transduced RGCs was determined by dividing the sum of neurite length by the respective RGC count and normalized to non-transduced cells. HIL6-induced neurite length is presented as fold change compared to DsRed-transduced RGCs. Significance of intergroup difference was evaluated using Student’s t-test. Expression of hIL6 and phosphorylation of STAT3 in hIL6-baculovirus transduced RGCs was evaluated upon immunohistochemistry with antibodies against GFP (1:1000, NB100-1770, Novus Biologicals), IL-6 (1:500, ab6672, Abcam) and pSTAT3 (1:200, 9145 S, Cell Signaling Technology).

## Results

### Transduction of DRG neurons

**Figure 1:**
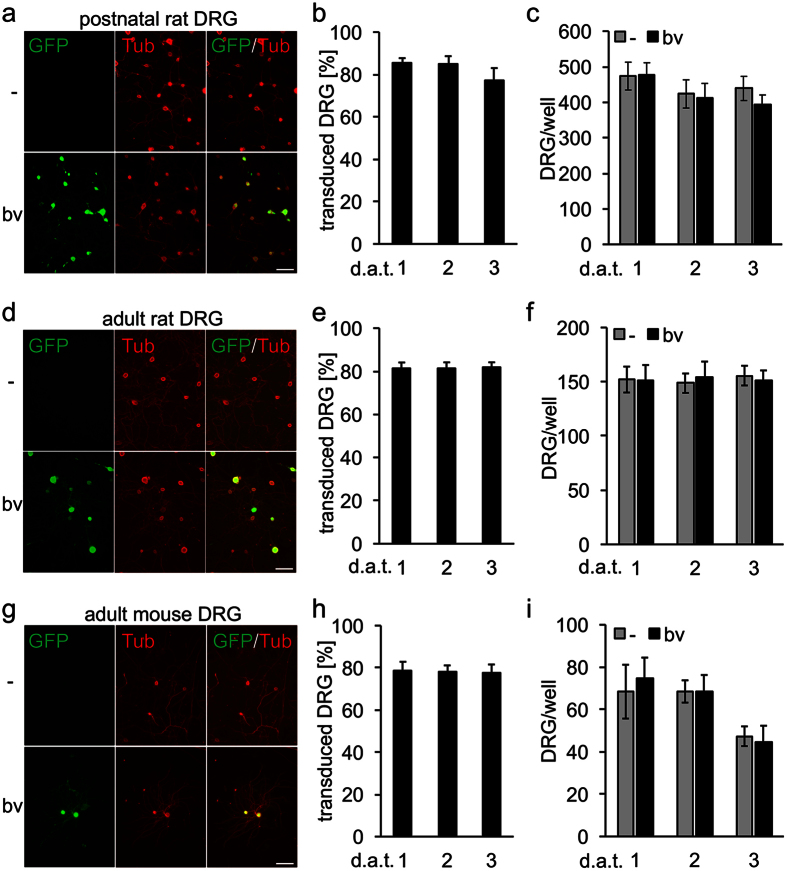
Transduction of dorsal root ganglion neurons. Dissociated dorsal root ganglion neurons (DRG) isolated from postnatal (**a**–**c**) or adult (**d**–**f**) rats and adult mice (**g**–**i**) were transduced with fGFP-baculovirus (bv). Representative pictures of vehicle (-) and bv -treated cultures (**a**,**d**,**g**) show transduced, GFP-expressing neurons (green) that were co-stained with the neuronal marker βIII-tubulin (Tub, red) at 2 days after transduction (d.a.t.). The percentage of transduced, GFP-expressing DRG neurons was determined at 1, 2 and 3 d.a.t. (**b**,**e**,**h**), revealing similar transduction efficiencies of 80–90% in postnatal and adult DRG neurons. Cell survival in postnatal (**c**) and adult (**f**, **i**) DRG cultures was not affected by bv-application compared to vehicle-treated controls (-). Scale bars: 100 μm.

Gene transfer generally tends to be more effective with younger primary neurons. Accordingly, baculovirus (bv)-mediated transduction of embryonic and postnatal neurons was reported with 20–30% efficiency^23^,^24^,^25^,^26^. Therefore, we first tested our BacMam preparations on dorsal root ganglion (DRG) neurons isolated from P7 rat pups. Virus particles were directly added to dissociated DRG neurons 4 h after seeding, ensuring sufficient time for cell attachment. The proportion of transduced (GFP-expressing) and βIII-tubulin-positive neurons was determined at 1, 2 and 3 days after transduction (d.a.t.). This approach reliably achieved transduction rates of ~80% for postnatal DRG neurons (Fig. 1a,b). Of note, no differences were observed for the different time points analyzed, indicating efficient transduction and fast induction of recombinant protein expression within 24 h. Despite these high transduction rates, counts of DRG neurons did not differ between vehicle- and bv-treated cultures (Fig. 1c), indicating low neurotoxicity of our BacMam preparation.

Based on this promising result with postnatal neurons, we also applied baculovirus particles to mature neuronal cultures. Strikingly, similar transduction efficiencies (~80%) were observed for DRG neurons isolated from adult rats (Fig. 1d,e) as well as adult mice (Fig. 1g,h), without any apparent signs of neurotoxicity (Fig. 1f,i). GFP expression was strong already at 1 d.a.t., allowing easy visualization of axonal processes. Therefore, BacMam-mediated gene delivery is an easy-to-use and reliable method to efficiently express heterologous proteins in cultured postnatal as well as adult peripheral sensory neurons of different mammalian species.

### Transduction of mature retinal ganglion cells

**Figure 2:**
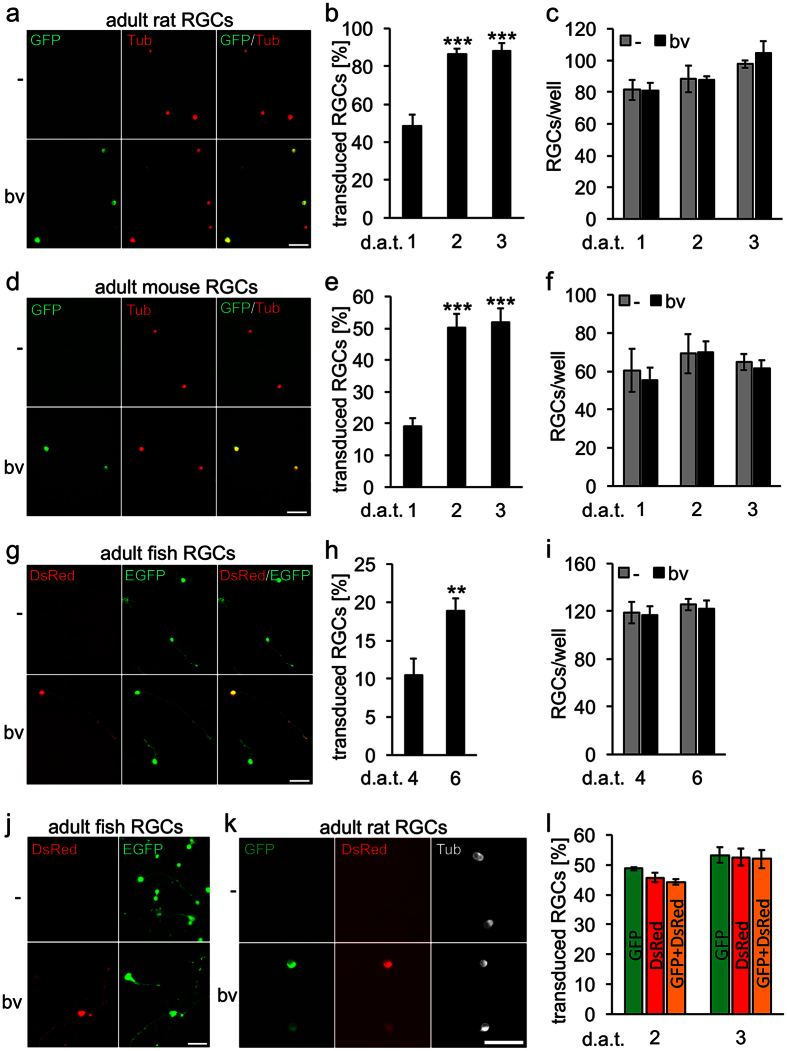
Transduction of mature retinal ganglion cells. Dissociated retinal ganglion cells (RGCs) isolated from adult rats (**a**–**c**), mice (**d**–**f**) and zebrafish (**g**–**i**) were transduced with baculovirus (bv) encoding fGFP (rat and mice) or DsRed (zebrafish). Representative pictures of vehicle- (-) and bv -treated cultures (**a**,**d**,**g**) visualize transduced RGCs (GFP and DsRed, respectively) that were either co-stained with the neuronal marker βIII-tubulin (Tub, red) at 2 days after transduction (d.a.t) for rat and mice RGCs (**a**,**d**) or identified by EGFP expression for zebrafish RGCs at 6 d.a.t. (**g**). The percentage of transduced RGCs was determined at 1, 2 and 3 days after transduction (d.a.t.) for rat and mice (**b**,**e**) and at 4 and 6 d.a.t. for zebrafish (**h**). Treatment effects compared to vehicle-treated controls: ***p < 0.001, **p < 0.01. Scale bars: 50 μm. RGC survival (**c**,**f**,**i**) was not affected by baculovirus application (bv) compared to vehicle-treated controls (-). (**j**) Delayed transduction of adult zebrafish RGCs with DsRed-bv after 4 days in culture. Scale bar: 50 μm. (**k**,**l**) Co-transduction of adult rat RGCs with fGFP-bv and DsRed-bv. The two viruses were added to retinal cultures simultaneously at half the concentration of single transductions. Representative pictures show co-transduced, fGFP- and DsRed-expressing RGCs (green and red, respectively) that were co-stained against the neuronal marker βIII-tubulin (white) at 2 d.a.t. Scale bar: 50 μm. (**k**). The percentage of transduced RGCs was determined at 2 and 3 d.a.t. (**l**). Non-significant difference in co-tranduction efficiency compared to single transductions.

Primary mature CNS neurons are usually very difficult to culture, but retinal ganglion cells (RGCs) isolated from adult mammals can be kept in culture for up to 5 days prior to the onset of neuronal degeneration^21^. These neurons are therefore frequently used for the study of age dependent processes, such as neuronal survival and axon regeneration^22^,^27^,^28^. However, efficient and fast *in vitro* gene transfer is, to our knowledge, still virtually impossible and hampers experimental approaches. Therefore, we next investigated whether adult RGCs would be equally amenable to BacMam transduction as DRG neurons. To this end, retinal cells isolated either from adult rats or mice were incubated with GFP-encoding BacMam virus (Fig. 2). GFP fluorescence became clearly visible within 24 h in βIII-tubulin positive RGCs, indicating efficient transduction and protein synthesis. The transduction rate for rat RGCs increased from ~50% at 1 d.a.t. to almost 90% at 2 and 3 d.a.t. (Fig. 2a,b). Transduction of mouse RGCs was less extensive, but still substantial with ~20% at 1 d.a.t. and ~50% at 2 and 3 d.a.t. (Fig. 2d,e). The number of RGCs was unchanged upon viral transduction compared to vehicle-treated controls (Fig. 2c,f), again demonstrating low cytotoxicity. To our knowledge, this is the first report of efficient gene transfer into cultured adult mammalian RGCs. The fact that maximal transduction rates were only achieved delayed compared to DRG neurons suggest general slower induction of exogenous protein expression in RGCs.

Zebrafish RGCs have recently been established for the analyses of regeneration competent CNS neurons^22^, but methods for their genetic manipulation are even more sparse than for mammalian neurons. As baculoviruses can reportedly transduce embryonic zebrafish cells *in vivo*^29^, we also analyzed BacMam-mediated transduction of dissociated retinal cells isolated from adult zebrafish. Induction of DsRed transgene expression in zebrafish RGCs was considerably slower compared to mammalian RGCs and was detected in ~10% RGCs at 4 d.a.t. and ~20% at 6 d.a.t (Fig. 2g,h), without neurotoxic effects on cell counts. These varied proportions of transduced RGCs in rat, mice and zebrafish indicate species-specific differences in BacMam transduction rates. Some experimental approaches might favor gene transfer after extended culturing time, for example when neurons have already extended neurite processes. In order to test this possibility, BacMam virus was added to zebrafish RGCs after 4 days in culture (Fig. 2j). Remarkable, the transduction rate was with ~18% comparable to immediate virus application. Thus, neurons cannot only be efficiently transduced immediately after plating, but also several days later, leaving some leeway for the initiation of recombinant protein expression within a given experiment.

It is often desirable to induce expression of more than one heterologous protein in a neuron (e.g. the gene of interest and a fluorescent marker protein). Therefore, we also analyzed co-transductions with two BacMam viruses encoding different fluorescent marker proteins (GFP and DsRed) (Fig. 2k,l). Halving each virus concentration (~5× 10^5 ^pfu each) compared to previous experiments, respective single transduction rates were expectedly reduced. Now, ~50% rat RGCs expressed either GFP or DsRed at 2 and 3 d.a.t. (Fig. 2l). As visible DsRed expression took longer to appear than for GFP, transduction rates were not analyzed at 1 d.a.t. in this experiment. Strikingly, almost every transduced RGC was double-positive for both marker proteins, indicating efficient and reliable co-transduction (Fig. 2k,l). All in all, adult RGCs across different species were readily transduced by BacMam viruses and the options of delayed gene transfer and virtual co-transduction might be advantageous for functional *in vitro* assays, facilitating novel experimental options.

### Induction of cre-mediated recombination

**Figure 3:**
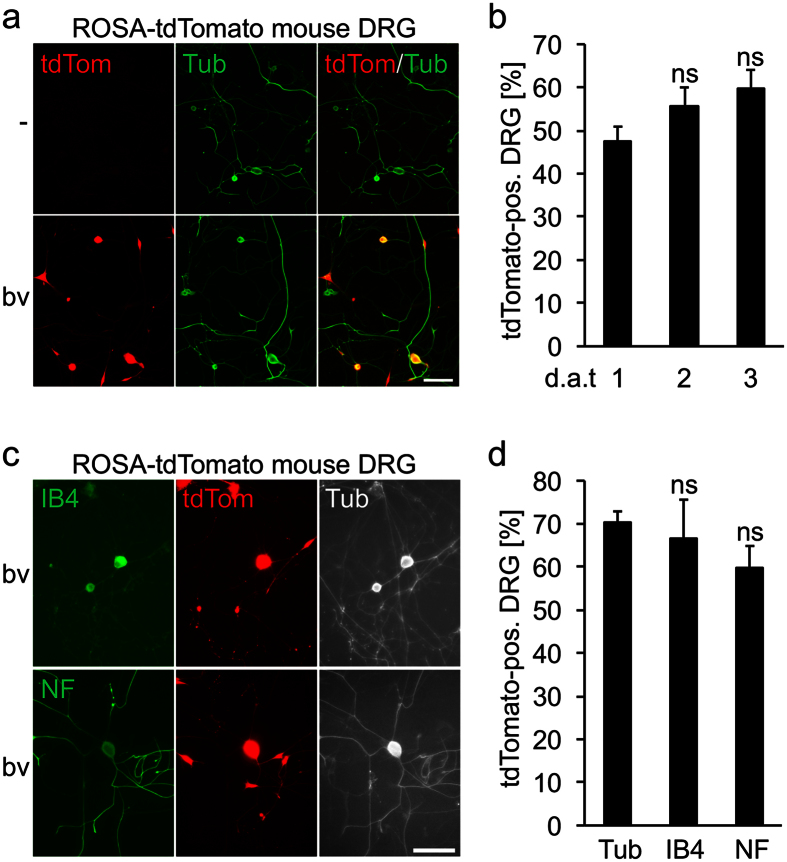
Induction of cre-mediated recombination. Adult dorsal root ganglion neurons (DRG) isolated from ROSA-tdTomato mice were transduced with baculovirus encoding cre recombinase (cre-bv) (**a**–**d**). Representative pictures of cre-bv-treated cultures (**a**,**c**) show transduced, tdTomato-expressing DRG (red) that were co-stained against the neuronal markers (green) βIII-tubulin (Tub) (**a**), neurofilament- (NF) and isolectine B4- (IB4) (**c**) at 3 days after transduction (d.a.t). The percentage of transduced cells in relation to βIII-tubulin was determined at 1, 2 and 3 d.a.t. with non-significant (ns) differences compared to 1 d.a.t. (**b**). In addition, the proportion of transduced NF- and IB4- positive DRG subpopulations was determined (**d**), revealing non-significant (ns) differences in transduction efficiencies compared to βIII-tubulin-positive neurons Scale bars: 100 μm.

In further experiments, we wanted to ensure the expression of functional heterologous proteins upon BacMam virus transduction using cre-mediated recombination and neurite outgrowth assays. The cre/loxP- system is widely used for targeted, conditional knockout experiments, which for one depends on efficient and timely cre recombinase expression, particularly in cell cultures. However, bv-mediated expression of cre recombinase in postnatal mouse striatal neurons was reportedly insufficient to induce genetic recombination, although the same construct was active in neuronal cell lines^30^. We therefore tested a BacMam virus encoding cre recombinase on adult DRG neurons isolated from ROSA-dtTomato mice. Expression of dtTomato indicates successful recombination upon induced enzymatic cre activity (Fig. 3a) and was detected in roughly 50% DRG neurons at 1 d.a.t., increasing to ~60% at day 3 (Fig. 3b). Therefore, cre-mediated recombination is quickly and efficiently initiated upon BacMam-transduction, which should enable the analysis of target gene-specific knockouts in primary neuronal cultures.

In separate experiments, we additionally quantified the transduced proportions of DRG subpopulations, which can be histologically classified by different marker expression (Fig. 3c,d). For instance, non-peptidergic small-diameter neurons bind isolectin B4 (IB4), while large-diameter neurons are immunoreactive for neurofilament heavy chain (NF). Overall, a transduction rate of ~70% was determined for all, βIII-tubulin-positive DRG neurons at 3 d.a.t. (Fig. 3d), which is comparable to transduction with GFP-encoding BacMam virus (Fig. 1). Similar (60–70%), not significantly different percentages were detected for NF- and IB4- positive neurons, indicating that different DRG subpopulations are equally efficiently transduced (Fig. 3d). Therefore, BacMam-mediated cre expression is functional in adult cultured neurons and does not show any preference for specific cell types.

### Induction of hyper-IL6- mediated neurite growth

**Figure 4:**
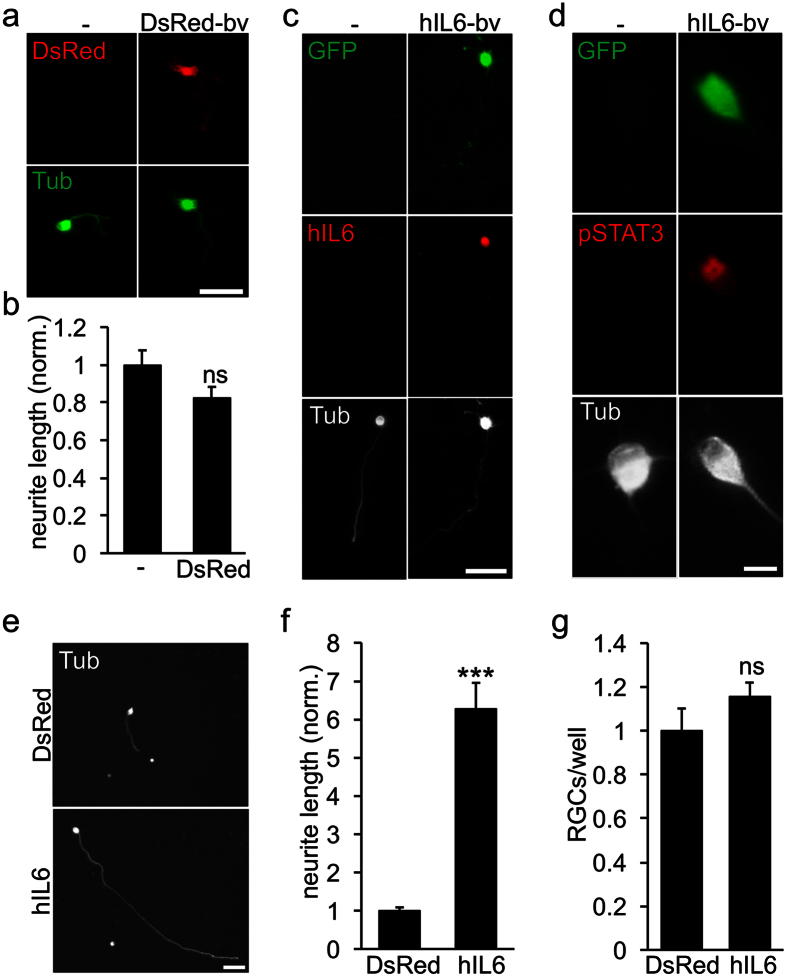
Induction of hIL6- mediated neurite growth. Adult mouse retinal cells were transduced either with control DsRed-bv (**a**,**b,e**–**g**) or hIL6-bv (baculovirus encoding hIL6 and EGFP) (**c**–**g**). (**a**) Representative pictures show vehicle- (-) and DsRed-bv-transduced retinal ganglion cells (RGCs) stained with the neuronal marker βIII-tubulin (Tub, green) at 4 days after transduction (d.a.t.). (**b**) Quantification of mean neurite length normalized to vehicle-treated controls (-) with an average neurite length of 20.3 μm/RGC reveal no generalized effect of control baculovirus on neurite outgrowth. Representative pictures of vehicle- (-) and hIL6-bv-treated RGCs at 4 d.a.t. (**c**,**d**) show expression of hIL6 (**c**) and induction of STAT3 phosphorylation (**d**) in transduced, GFP-expressing (green) RGCs that were co-stained against βIII-tubulin (white). Representative pictures of RGC neurite growth upon DsRed-bv or hIL6-bv transduction (**e**) and quantification of mean neurite length normalized to DsRed-bv-transduced RGCs with an average value of 23.1 μm/RGC (**f**) at 4 d.a.t. illustrate ~6-fold longer, βIII-tubulin-positive neurites upon hIL6 expression. Treatment effect: ***p < 0.001 (**g**) RGC survival was similar for both baculoviruses. ns = non-significant. Scale bars: 50 μm in a, c and e; 10 μm in d.

Application of the designer cytokine hyper-IL-6 (hIL6) activates the janus kinase/signal transducer and activator of transcription 3 (JAK/STAT3) pathway in cultured RGCs and markedly promotes their neurite growth^19^,^31^. We adapted this approach to further evaluate the functionality of heterologous proteins expressed upon BacMam-mediated gene delivery and to demonstrate the feasibility of assaying the role of a specific protein in established *in vitro* neurite outgrowth assays via viral-induced expression. To this end, DsRed-encoding control or hIL6-encoding BacMam virus was applied to mouse retinal cultures at 4 h after plating (Fig. 4). Quantification of mean RGC neurite length at 4 d.a.t revealed no significant difference for DsRed-bv compared to vehicle-treated cultures (Fig. 4a,b). This result, together with unchanged RGC numbers (Fig. 2f), confirmed neuronal tolerance for BacMam transduction. Expression of the designer cytokine hIL6 was expectedly detected in transduced, GFP-positive RGCs, but not in control cultures (Fig. 4c). Functional activity of hIL6 was established through increased phosphorylation of STAT3 in transduced RGCs (Fig. 4d). In addition, RGC neurite growth was ~6-fold longer upon hIL6 expression compared to DsRed-bv-transduced control RGCs (Fig. 4e,f). Again, no neurotoxicity was observed, as RGC survival was similar for both BacMam viruses (Fig. 4g). Therefore, active, recombinant proteins can be swiftly and efficiently expressed in adult neurons upon BacMam transduction, enabling versatile overexpression and knockdown studies in cultured neurons in the future.

## Discussion

The current study provides a highly efficient, reliable and fast method to transfer recombinant DNA into adult primary neurons directly in culture. Although transduction/transfection of embryonic and postnatal neurons by various methods has been described previously, respective reports for primary adult neurons are still missing. Consistently, our own experience has shown that most approaches are not straightforwardly transferred to adult neurons. However, research aspects relating to the adult CNS or PNS are best examined using mature neurons. One example is regenerative axonal growth, which crucially depends on neuronal age^32^,^33^. Using the recombinant BacMam system, we could now demonstrate for the first time that mature neurons from the PNS (DRG neurons) as well as from the CNS (RGCs) of various species can be readily transduced with high efficiency in culture. Highly reproducible transduction rates of ~80% were detected for rat and mice adult DRG neurons and different neuronal subpopulations were transduced to similar extent without obvious preferences. In comparison, some species specificity was observed for RGCs. Whereas transduction rates of ≥ 80% were also observed for rat RGCs, ~50% mouse RGCs were transduced with the same viral stock under comparable culture conditions. Although it might be possible to further increase this percentage by applying higher MOIs, the achieved efficiency was more than adequate for our *in vitro* experiments. In fact, assay interpretation might be even more reliable if transduced and untransduced cells can be evaluated in the same well. Lowest efficiency of ~20% was established for zebrafish RGCs, which would still be sufficient to quantify the impact of heterologous protein expression^22^,^28^. In fact, this is to our knowledge the first report of successful gene transfer into primary zebrafish neurons in culture. All in all, unexpectedly high gene transfer rates were observed for all tested neurons, although variability between neuronal types and between species was noted, which may require individual pre-testing in new experimental settings.

Transduction of primary adult neurons was straightforward and simple, as viral inoculum was just added directly to neuronal cultures. Surprisingly, high transduction rates were observed without any application of histone deacetylase inhibitors such as butyrate, which is often used to increase baculoviral gene transfer and recombinant protein expression^10^,^12^. As this treatment was deemed unnecessary for our purposes, inhibitor-induced toxicity was avoided. Generally, baculoviruses are known for their low cytotoxicity and superior biosafety profile. Accordingly, numbers of surviving neurons in our cultures did not change over time compared to untreated controls. However, a partial change of culture medium at 10–20 h after virus application was essential to avoid a partial loss of cells. In addition, progressive loss of neurite processes was noted upon strong fGFP expression, as DRG axons became fragmented after 3 days in culture. Potentially, extensive intercalation of fGFP into the plasma membrane was detrimental to cultured neurons. This is consistent with previously reported EGFP-induced apoptosis^34^, but was not observed upon monomeric DsRed expression. Nevertheless, MOI rates were reduced for all outgrowth assays in our study to elude any potential negative side effects.

In accordance with the apparent indiscrimination of baculovirus hosts, transgene expression was also detected in some non-neuronal cells in our mixed cultures. However, their proportion was rather low, potentially due to the fact that our culture conditions favored the survival of neurons. Their presence did not interfere with assay evaluation, as they were clearly distinguishable upon neuronal marker staining. Alternatively, neuron-specific regulatory elements could be inserted into the viral vectors to drive gene expression exclusively in neurons^25^,^35^. Fluorescent marker expression became already evident in rat and mice neurons within a few hours after transduction, although DsRed took slightly longer and maximum transduction levels required 2 days in RGCs. Nevertheless, this time course is considerably faster compared to LV-transduction, which takes 2–5 days in rat DRG neurons^36^ and is even slower for AAVs^2^. Due to the limited survival time of most mature neurons in culture, slow induction of transgene expression might, however, not leave enough time to evaluate certain parameters, such as effects on neurite growth. For this reason, AAVs are generally not applied *in vitro*, but are injected *in vivo* several weeks prior to culture preparation^27^. Depending on the experimental setting, it might, however, be advantageous to analyze the immediate effects of heterologous protein expression without prolonged pre-treatment, as this could already impact neural physiology. This approach is now possible using recombinant BacMam viruses. Exemplary, we confirmed neurite growth-promotion upon BacMam-mediated hIL6 expression in cultured RGCs. Overall, this protocol is considerably more time- and cost-efficient compared to *in vivo* AAV transduction and achieved at least similar, if not better growth promotion (compare to ref. 19). The early onset of gene expression also enabled cre-mediated genome editing *in vitro*. In contrast to a previously reported unsuccessful attempt^30^, we observed fast and efficient recombination in adult neurons. Thus, conditional gene knockout studies are possible with this approach, considerably extending experimental capabilities in culture.

Construction of recombinant BacMam virus is relatively easy, mostly requiring only a biosafety level 1 facility. Either purified virus or infected cells can be stored almost indefinitely^37^ and high titers can be recurrently and reproducibly produced simply by re-infection of insect cells, hence providing ongoing supply at low effort. Another advantage is the large cloning capacity of baculovirus (up to 38 kbp) compared to LV- and AAV-vectors, facilitating expression of larger proteins and even multicomponent protein complexes, for example for crispr/cas-mediated genome engineering^10^,^12^,^13^. Alternatively, co-expression of several cDNAs can be achieved upon baculovirus co-transduction. Conveniently, virtually the same neurons were transduced upon application of two BacMam viruses, similar to previously described AAV-mediated transduction *in vivo*^38^. The prevalently transient nature of BacMam-mediated expression should not pose a drawback for neurons and avoids the risk of insertional mutations. Most assays using primary neurons are anyway completed within a few days. In addition, BacMam-mediated expression might even persist longer in differentiated neurons compared to mitotic cells, as cell-division-induced dilution does not occur. Accordingly, no decline in marker expression was detected up to 8 days in zebrafish RGC cultures (data not shown). Alternatively, transgene expression might be extended by means of BacMam re-application at later stages^39^, as neurite-bearing neurons were also readily transduced. All in all, we consider the efficient BacMam-mediated gene transfer into cultured neurons a very versatile approach for a wide variety of applications in neurobiology research.

## Additional Information

**How to cite this article**: Levin, E. *et al*. Highly efficient transduction of primary adult CNS and PNS neurons. *Sci. Rep.* **6**, 38928; doi: 10.1038/srep38928 (2016).

**Publisher's note:** Springer Nature remains neutral with regard to jurisdictional claims in published maps and institutional affiliations.
